# Editorial: Biofilms: multi-species community interactions

**DOI:** 10.3389/fmicb.2023.1191406

**Published:** 2023-05-25

**Authors:** Sanket J. Joshi, Rina Rani Ray, Garth D. Erlich, Hisham A. Edinur

**Affiliations:** ^1^Oil & Gas Research Center, Central Analytical and Applied Research Unit, Sultan Qaboos University, Seeb, Oman; ^2^Department of Biotechnology, Maulana Abul Kalam Azad University of Technology, Kolkata, India; ^3^College of Medicine, Drexel University Philadelphia, Philadelphia, PA, United States; ^4^School of Health Sciences, Universiti Sains Malaysia, Kubang Kerian, Kelantan, Malaysia

**Keywords:** biofilm communities, antibiotic resistance, virulence, quorum sensing, autoinducers

Biofilms are a group of adherent sessile bacterial colonies that form a syntrophic consortium. Multispecies biofilm communities have been observed in both biotic and abiotic habitats, including the human body (Lahiri et al., 2021a). Besides bacteria, fungi and algae are also known to develop biofilm (Limoli et al., 2015). The formation of biofilm provides shelter for microbial colonies from various types of environmental stress and it is associated with 60–80% of pathogenic infections. The development of biofilm acts as an important virulence factor, especially for microbe species that can evade the host immune response (Dutta et al., 2021). Genetic alterations also crucial in the development of virulence as well as antibiotic resistance (Lahiri et al., 2021b). The multifactorial nature of biofilms may enhance the resistance of bacteria to conventional drugs, thereby creating a challenge in the use of antibiotics.

Acute infections are presumed to be caused by planktonic bacteria and can effectively be treated with antibiotics (Ray et al., 2021). However, in certain chronic cases, microbial cells aggregated into biofilms and are resistance to conventional medical treatments. The mechanism of biofilm formation varies from species to species, but the common feature is the extracellular polymeric substances (EPS) that holds the cells together. Quorum sensing (QS) is the ability of bacteria to sense cell density and synchronize their behavior through cell-to-cell signaling using small molecules known as autoinducers (Ais) (Nag et al., 2021). These Ais will cause biofilm-forming cells to aggregate with one another through secretion of EPS, which are majorly responsible for the expression of virulence in pathogens. Thus, the contemporary challenge is to understand how QS works in the host environment that make antimicrobial susceptibility studies becoming complex, especially with the absence of a consensus or standardized protocols. Armed with the basic understanding on how biofilms facilitate QS and protect the microbial community from antibiotics, many researchers have studied various compounds and substances with the aim of inhibiting biofilm formation, or at least disrupt the interaction between bacteria to reduce their pathogenicity.

In this Research Topic, Sun et al. evaluated the inhibitory effects of esculetin (a plant phenolic compound known as hydroxylated coumarin) on QS and biofilm formation of gram-negative bacteria (i.e., *Aeromonas hydrophila*). Their findings indicated that esculetin could inhibit the swarming motility of *A. hydrophila* and its biofilm formation as determined through confocal laser scanning microscopy and scanning electron microscopy. These were further supported by the reduced expression of genes related to QS and biofilm formation as assessed by quantitative RT-PCR. In another study, Jiang et al. examined the effects of compound Str7410, an autoinducer-2 (AI-2) inhibitor, on interspecies QS *in vitro* and *in vivo*. The authors observed that a co-culture of *Pseudomonas aeruginosa* and *Streptococcus aureus*, when treated with a combination of Str7410 and the antibiotic meropenem trihydrate, could increase the susceptibility of biofilm cells to the antibiotic, besides inhibiting AI-2 signaling and downregulating the expression of QS-related genes in *P. aeruginosa*.

Another related study on QS was reported by Beenker et al. This study aimed to examine the effects of secondary metabolites from a fungus in inhibiting QS. They reported that the gregatin family of compounds could inhibit QS in *P. aeruginosa*, but not biofilm formation. An interesting study was reported by Jarzynka et al. on the effects of human milk oligosaccharides (HMOs) on biofilm eradication activities. Many earlier studies had been using *B. streptococci* as a model to assess the antimicrobial activity of HMOs. However, antimicrobial activity of HMOs in this study was investigated using a wide spectrum of bacteria, comprising seven gram-positive and gram-negative species. Their findings showed that antimicrobial activity of HMOs was mostly effective on gram-positive species. They also managed to identify fucosyllactose as the antibacterial component in HMOs.

Finally, Flores-Vargas et al. wrote a review describing how natural river biofilms may act as reservoirs of antibiotic resistance. Even though the threat of antimicrobial resistance is higher in hotspots like hospitals and farms, the authors cautioned that downstream environments like lakes and rivers are also constantly exposed to sub-inhibitory levels of antibiotics, which applies pressure for the selection of resistant bacteria. However, the extent of how the low concentrations of contaminating antibiotics drive the proliferation of resistance in a natural environment is not fully understood. This mechanism is further complicated by the role of bacteriophages found among the biofilm microorganisms.

In conclusion, collection of articles in this Research Topic would help the reader in getting new knowledge and research concepts pertaining to the role of biofilm in disease virulence and development of antibiotic resistance. These articles have attracted wide interest of biomedical community, scientists and medical practitioners, as demonstrated by 12,016 views, 3 citations and more than 1,350 downloads.

## Author contributions

All authors listed have made a substantial, direct, and intellectual contribution to the work and approved it for publication.
